# Diagnostic Value of Vaginal Ultrasound under Improved Clustering Algorithm Combined with Hysteroscopy in Abnormal Uterine Bleeding

**DOI:** 10.1155/2022/6951692

**Published:** 2022-05-27

**Authors:** Yuhui Wang, Qionghui Long

**Affiliations:** ^1^Department of Gynaecology and Obstetrics, The Fourth Hospital of Changsha, Changsha 410006, Hunan, China; ^2^Department of B-ultrasound Imaging, The Fourth Hospital of Changsha, Changsha 410006, Hunan, China

## Abstract

In order to explore the diagnostic value of the improved clustering algorithm of vaginal ultrasound combined with hysteroscopy in abnormal uterine bleeding (AUB), 128 patients diagnosed with AUB in the hospital were selected as the research objects. A *K*-means improved clustering color image segmentation algorithm was designed and applied to AUB vaginal ultrasound image processing. The running time, mean square error (MSE), and peak to signal noise ratio (PSNR) were calculated to evaluate the algorithm, and the sensitivity, specificity, negative likelihood ratio, and positive likelihood ratio were used to evaluate the diagnostic accuracy of the detection method. In addition, combined with hysteroscopy, a comprehensive evaluation of the diagnostic value of abnormal uterine bleeding diseases was implemented. The results showed that compared with the traditional *K*-means clustering algorithm, the running time of the improved *K*-means clustering color image segmentation algorithm in the training set was significantly shortened, the MSE was significantly decreased, and the PSNR was significantly increased (*P* < 0.05). The sensitivity, specificity, negative likelihood ratio, and positive likelihood ratio (90.5%, 93.2%, 84.3, and 96.3%) of AUB diagnosis were significantly improved in the algorithm of vaginal ultrasound combined with hysteroscopy (*P* < 0.05). In summary, the combination of vaginal ultrasound and hysteroscopy based on *K*-means improved clustering color image segmentation algorithm can significantly improve the clinical diagnostic accuracy of AUB patients.

## 1. Introduction

Abnormal uterine bleeding (AUB) refers to uterine bleeding under abnormal circumstances [1]. It can be caused by organic diseases, including systemic or reproductive changes, or iatrogenic factors such as intrauterine devices (IUD), the use of steroids, and contraceptives. It can also be caused by a functional disorder called dysfunctional uterine bleeding (DUB) [2, 3]. AUB has nine categories, including endometrial polyps, adenomyosis, endometrial hyperplasia, ovulation disorders, and abnormal coagulation function, which can all lead to abnormal uterine bleeding [4]. AUB is characterized by a disrupted menstrual cycle. Period length varies, the amount of bleeding is more or less, and there is even a lot of bleeding. Sometimes several weeks or months of menopause appear first, and then vaginal irregular bleeding occurs, and blood volume is often more, lasting 2–4 weeks or longer, and not easy to stop. Sometimes it starts with irregular vaginal bleeding or periodic bleeding similar to normal menstruation [5]. Hemorrhage period does not have lower abdomen ache or other unwell, hemorrhage much or time elder often companion anemia. Gynecological examination showed that the uterine size was in the normal range, and the uterus was soft when bleeding [6]. There are many reasons for abnormal uterine bleeding, which can be caused by systemic diseases, gynecological diseases, and iatrogenic factors. In addition, abnormal uterine bleeding that is not caused by organic disease is often referred to as dysfunctional uterine bleeding [7]. At present, clinical diagnostic examination methods for AUB include blood routine, sex hormone measurement, ultrasound examination, hysteroscopy, and diagnostic curettage [8]. Of which, ultrasound is mainly used to check whether there are changes in the endometrium, whether there are growth organisms in the uterine cavity, and whether there are abnormal masses in the ovary and bilateral appendices [9]. For local lesions of the endometrium, such as endometrial polyps or endometrial hyperplasia, hysteroscopy is required. Hysteroscopy can clarify the morphology of the uterine cavity and cervix and determine whether there are malformations, polyps, fibroids, or malignant space-occupying lesions in the uterine cavity [10]. In the process of hysteroscopy, the size of lesions and abnormal blood vessels can be observed, and the benign and malignant space-occupying diseases can be preliminary determined. In addition, targeted curettage can be performed according to the situation of hysteroscopy to prevent the occurrence of missed curettage and improve the diagnostic accuracy of diseases [11]. The early stage of endometrial cancer is limited to the endometrial mucosal layer or superficial base layer, but the site of occurrence is often found in the uterine horn, and the traditional curettage is easy to miss curettage, thus missing the diagnosis of endometrial cancer [12]. If hysteroscopy is adopted, the situation of the entire endometrial can be determined, so hysteroscopy can reduce the occurrence of curettage and leakage in traditional diagnosis and improve the accuracy of endometrial cancer diagnosis [13].

In recent years, image processing algorithms for various images emerge in an endless stream, such as deep learning algorithm, convolutional neural network algorithm, filtering algorithm, iterative algorithm, and clustering algorithm, which can improve image quality and image segmentation efficiency based on original image data, so as to improve the accuracy of image diagnosis of diseases [14]. The *K*-means clustering algorithm is an iterative clustering analysis algorithm. It divides the data into *K* groups, then randomly selects *K* objects as the initial cluster center, calculates the distance between each object and each seed cluster center, and assigns each object to the cluster center nearest to it. The *K*-means clustering is a Gaussian Mixture Model (GMM) solved with the expectation maximization algorithm. The covariance of the normal distribution is the unit matrix, and the posterior distribution of hidden variables is a special case of a set of Dirac delta functions [15]. The *K*-means algorithm is simple and efficient, but it is sensitive to initial parameters. Some studies obtained adaptive initial parameters by calculating intraclass variance to continuously adjust data classification and center position, or used a subtraction clustering algorithm to estimate the initial center position to improve the quality of segmentation results, but still rely on user input parameters [16]. Therefore, it was hoped to introduce the *K*-means improved clustering color image segmentation algorithm into the vaginal ultrasound image diagnosis process of patients with abnormal uterine bleeding and comprehensively evaluate the application potential of the algorithm by evaluating the algorithm performance and comparing the diagnostic accuracy, to evaluate the value of hysteroscopy combined with ultrasonography in the clinical imaging diagnosis of abnormal uterine bleeding.

## 2. Materials and Methods

### 2.1. Research Objects

In this study, 128 patients with AUB in the hospital from March 2018 to March 2020 were chosen as the research objects. All the patients underwent vaginal ultrasound and hysteroscopy. The age range of all patients was 22–59 years, the mean age was (38.73 ± 6.65) years, and the mean course of the disease was (4.27 ± 1.23) months. According to etiological classification, this experiment included 15 cases of uterine fibroids, 24 cases of uterine smooth muscle disease, 11 cases of systemic coagulation-related diseases, 15 cases of ovulation disorders, 7 cases of local endometrial abnormalities, and 49 cases of endometrial polyps. All the subjects or their family members agreed to sign informed consent and this study had been approved by the ethics committee of hospital.

The inclusion criteria of the patients were as follows: patients who met the clinical diagnostic criteria for AUB; patients who had no serious cardiovascular and cerebrovascular diseases or kidney diseases; patients who satisfied the indications for hysteroscopy or surgery; and patients who could offer the complete medical records and signed the informed consents.

The patients who met the following exclusion criteria were excluded: patients who had bleeding caused by cervical lesions or vulvovaginal lesions and patients who had bleeding caused by foreign bodies such as the intrauterine device.

### 2.2. Instruments and Procedures of Vaginal Ultrasound and Hysteroscopy

A color ultrasound diagnostic instrument was used for the vaginal ultrasound examination. Before the vaginal ultrasound examination, the patients were required to empty the bladder, and the bladder lithotomy position was taken. The vaginal probe with a frequency of 4–9 MHz was placed into the vagina of patients to observe the contour of the uterus in the pelvic cavity, the morphology of the endometrium, echo characteristics, the location and size of bleeding point, blood flow, and the boundary between endometrium and myometrium. It was also observed whether the endometrium was continuous from the internal cervical orifice to the fundus and whether the texture was uniform.

In hysteroscopy, the imaging hysteroscopy was used, and its parameter information is shown in Table 1.

During hysteroscopy, the lithotomy position was taken, and the vaginal speculum was placed after anesthesia. The hysteroscopy was then placed into the uterine cavity to screen the fundus, walls of the uterine cavity, cervical tubes, and in the both fallopian tubes. After that, samples were taken for pathological examination.

### 2.3. Cervical Cancer Lesion Segmentation Model under the Improved *K*-Means Clustering Color Image Segmentation Algorithm

The improved clustering color image segmentation algorithm under *K*-means was introduced to process vaginal ultrasound images. For the algorithm, the initial parameters of the *K*-means algorithm needed to be obtained at first, and the Hue-Saturation-Intensity (HSI) color space histogram adaptation was used. The multidimensional features of color, texture, and spatial coordinates of pixel points were used to measure the similarity among pixel points [16]. In this process, it was necessary to extract the texture features of the local directional pattern (LDP) of the image. The distribution features of multiple pixels were shown, to make them more suitable for human subjective vision [17].

The image segmentation process of the *K*-means algorithm was actually a clustering process among pixels on the characteristics of each pixel. First, the set of *m* sample data was set as *Q*, whose expression is shown in equation (1). The center point is represented by *D* and the *s* centers could be represented as {*D*_1_, *D*_2_, *D*_3_,…*D*_*s*_}. The Euclidean distance was used to represent the distance between two samples, and the expression is expressed in equation (2). Equation (3) gives the update method of the clustering center. Equation (4) represents the definition of the objective function *W*:

(1) $$\begin{array}{c} Q=\left\{Q_{1},Q_{2},Q_{3},\ldots Q_{m}\right\}, \end{array}$$

(2) $$\begin{array}{c} \text{Dist}\left(Q_{j},Q_{g}\right)={\left(Q_{j}-Q_{g}\right)}^{2}, \end{array}$$

(3) $$\begin{array}{c} m_{s}D_{s}=\sum_{Q\in D_{s}}Q, \end{array}$$

(4) $$\begin{array}{c} R=\sum_{j=1}^{s}\sum_{g=1}^{m_{g}}\text{Dist}\left(Q_{g},D_{j}\right). \end{array}$$

**Figure 1:**
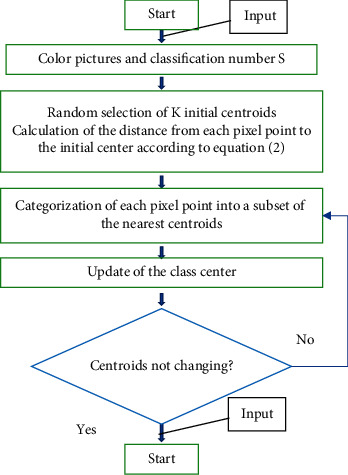
Image segmentation flowchart of the *K*-means algorithm.

In the above equations, *m*_*s*_ represents the number of sample points in the subset with the centroid *D*_*j*_ and *Q*_*g*_ is the sample points in the subset with the centroid *D*_*j*_. The flowchart of image segmentation of the *K*-means algorithm is shown in Figure 1.

**Figure 2:**
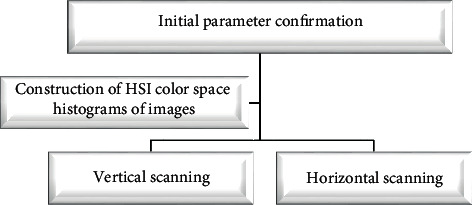
Flowchart of initial parameter confirmation of the *K*-means algorithm.

Then, the HSI color space, which was more in line with human visual perception, was used to identify the image color features of vaginal ultrasound images. In this process, the characteristic quantities of hue, saturation, and intensity were utilized to perceive the color of vaginal ultrasound images. It could not only simplify the workload of ultrasound image processing through independent component processing but also improve the color recognition accuracy of ultrasound images while reducing light sensitivity. The flow chart of the initial parameter confirmation of the *K*-means algorithm is shown in Figure 2, equation (5) gives the value range of the *K* during vertical scanning, and equation (6) shows the value range of the actual peak value.


(5) $$\begin{array}{c} V_{K_{j-1}}\leq V_{K_{j}}\geq V_{K_{j+1}}, \end{array}$$

(6) $$\begin{array}{c} K_{e}=\left(V_{K_{v}}\geq V_{K_{j}}\right)\forall V_{K_{j}}\in r. \end{array}$$

In the above two equations, K represents the color feature value obtained by quantizing the color, V represents the total number of pixels in each color interval, and *j*=1,2 …, *M*. *K*_*e*_ represents the actual peak point, and r represents the radius of each point in the peak set from the peak point.

Finally, in the calculation of the similarity of multidimensional features, feature vectors of different dimensions were used. The dimensions included the spatial coordinates of each pixel point (*X*, *Y* two-dimensional spatial coordinates), color (three parameters of hue, saturation, and intensity), and LDP texture features. The feature vector of each pixel point could be expressed as equation (7). The Euclidean distance of the multidimensional feature vector was used to represent the similarity distance among pixels, and the expression is shown in equation (8):

(7) $$\begin{array}{c} Z_{j}=\left(X_{j},Y_{j},H_{j},S_{j},I_{j},L_{j},\right), \end{array}$$

(8) $$\begin{array}{c} \text{Dist}\left(Q_{j},Q_{g}\right)={\left(Q_{j}-Q_{g}\right)}^{2}. \end{array}$$

Z stands for the number of pixels in the vaginal ultrasound image, *Z*_*j*_ represents any pixel, and *j*=1,2 …, *M*. *H*_*j*_, *S*_*j*_, and *I*_*j*_ represent the hue feature, saturation feature, and intensity feature of each pixel, respectively. L is the texture feature of the pixel. *X*_*j*_ and *Y*_*j*_ was the spatial coordinate of the pixel.

### 2.4. Quality Evaluation of Vaginal Ultrasound Images Processed by the Improved *K*-Means Clustering Color Image Segmentation Algorithm

The mean square error (MSE) and peak-to-signal noise ratio (PSNR) indicators were adopted to evaluate the similarity between the segmentation results of the improved clustering algorithm and the benchmark results. The mathematical expressions of MSE (the value range was [0, 1]) and PSNR are shown in equations (9) and (10), respectively:

(9) $$\begin{array}{c} \text{PSNR}\left(M,N\right)=10{\text{log}}_{10}\left[\frac{255^{2}}{\text{MSE}\left(M,N\right)}\right], \end{array}$$

(10) $$\begin{array}{c} \text{MSE}\left(M,N\right)=\frac{\sum_{p=0}\sum_{q=0}{\left[M\left(p,q\right)-N\left(p,q\right)\right]}^{2}}{AB}. \end{array}$$

In the above equations, *A* and *B* stand for the number of rows and columns of the input image, respectively. *M* represents the original image, and *N* represents the segmented image.

### 2.5. Analysis of Imaging Diagnosis Effect under the Improved *K*-Means Clustering Color Image Segmentation Algorithm

The diagnostic results from vaginal ultrasound images of AUB patients and the actual pathological results were compared and analyzed, as the images were processed by the traditional *K*-means algorithm and the improved *K*-means clustering color image segmentation algorithm, respectively. The sensitivity, specificity, negative likelihood ratio, and positive likelihood ratio were adopted in the comparison and analysis, to assess the diagnostic accuracy of the detection methods.

### 2.6. Statistical Methods

Statistical software SPSS19.0 was used to process the experimental data. The measurement data were expressed as mean ± standard deviation ($\bar{x}\pm s$), and the mean comparison between groups was performed by *t* test. The enumeration data were expressed by percentage, and the *χ*^2^ test was used. *P* < 0.05 indicated that the difference was statistically significant.

## 3. Results

### 3.1. General Information of Patients

**Figure 3:**
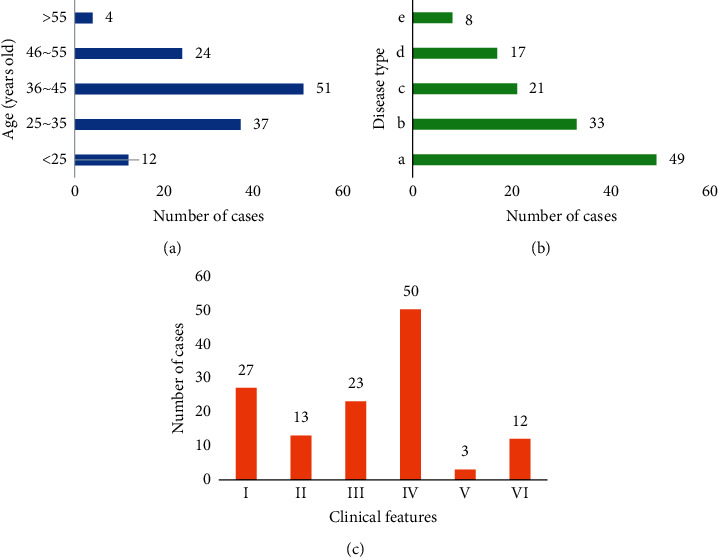
Analysis of the general data of the patients. (a–c) The age distribution, disease type distribution, and clinical characteristics distribution of the patients, respectively.

Figure 3 displays the analysis charts of the general data of the patients. It could be found from the figure that among the 128 AUB patients included, the majority of patients were at the age of 25–55 years old, out of which the largest number of patients were in the age of 36–45 years old. According to the types of diseases diagnosed by pathology, there were 49, 33, 21, 17, and 8 patients who suffered from endometrial hyperplasia, endometritis, endometrial polyps, uterine fibroids, and cervical and intrauterine adhesions, respectively. According to the clinical characteristics of the patients, 27 patients had irregular menstruation, 13 patients had prolonged menstruation, 23 patients went with increased menstrual volume, 50 patients had profuse vaginal bleeding, 3 patients had contact bleeding, and 12 patients had postmenopausal bleeding.

### 3.2. Results of Algorithm Performance Test

**Figure 4:**
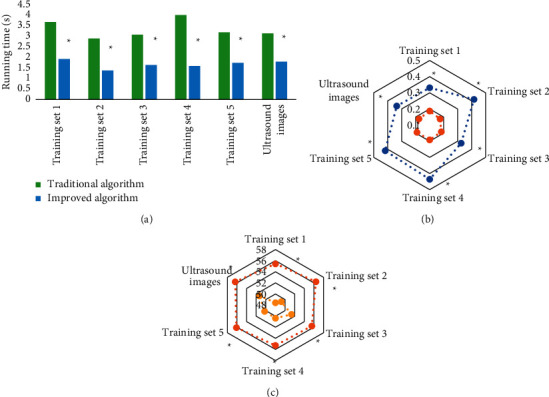
Comparison of the running time before and after the improvement of the *K*-means clustering color image segmentation algorithm in training sets. (a–c) The comparison of running time, MSE, and PSNR, respectively, before and after the improvement of the K-means clustering color image segmentation algorithm. ^*∗*^means the differences were of statistical significance compared with the traditional algorithm (*P* < 0.05).

Figure 4 shows the comparison charts of the running time in each training set before and after the improvement of the *K*-means clustering color image segmentation algorithm. It was suggested that, compared with that of the traditional *K*-means clustering algorithm, the running time of the improved algorithm in the training sets 1, 2, 3, 4, and 5 and clinical vaginal ultrasound images were all significantly shortened. The MSE value was significantly decreased, and the PSNR value was significantly increased, showing a statistically significant difference (*P* < 0.05).

### 3.3. Analysis of Vaginal Ultrasound Images of AUB Patients under the *K*-Means-Based Improved Clustering Color Image Segmentation Algorithm

**Figure 5:**
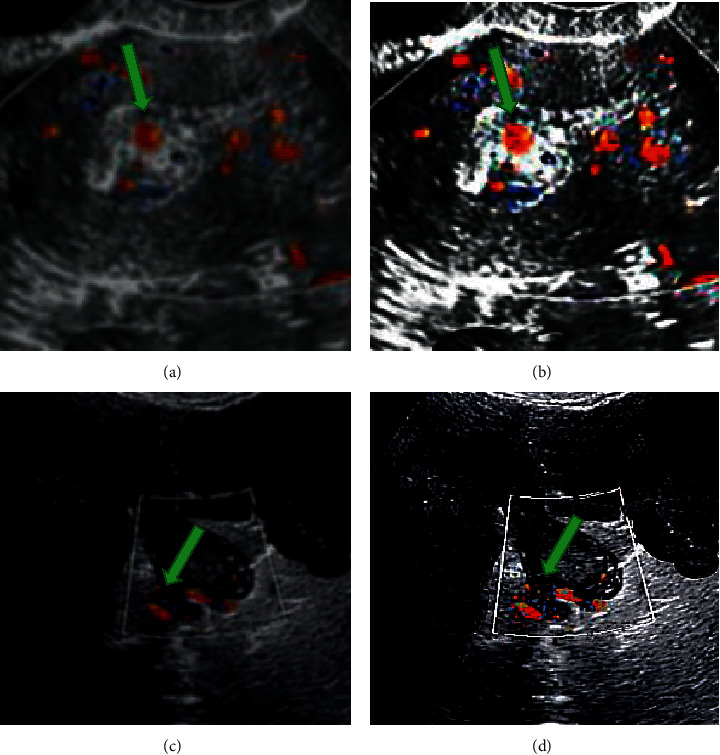
Vaginal ultrasound images of patients with AUB before and after being processed by the improved *K*-means clustering color image segmentation algorithm. (a, c) The original vaginal ultrasonography of patients A and B with abnormal uterine bleeding, respectively. (b, d) The vaginal ultrasound images of patients A and B with abnormal uterine bleeding processed by the *K*-means improved clustering color image segmentation algorithm, respectively.

Figure 5 shows the vaginal ultrasound images of patients with AUB before and after processing by the *K*-means-based improved clustering color image segmentation algorithm. It was observed from the images that after the images were processed by the improved *K*-means clustering color image segmentation algorithm, the visual texture of the patients' vaginal ultrasound images became clearer. The color segmentation of each pixel point was also clearer, and the contour imaging of the lesion position in the patients' uterus turned out to be more continuous.

### 3.4. Diagnostic Evaluation of Hysteroscopy and Vaginal Ultrasound of AUB Patients

**Figure 6:**
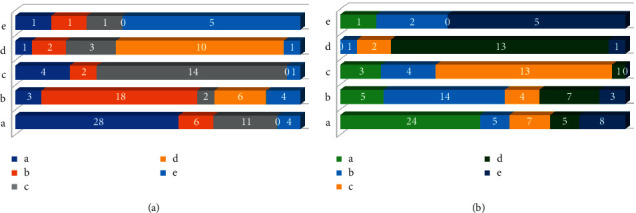
Comparison of results of hysteroscopy, vaginal ultrasound, and pathological examinations: (a) the comparison between hysteroscopy and pathological examination; (b) the comparison between vaginal ultrasound and pathological examination. a, b, c, d, and e represent endometrial hyperplasia, endometritis, uterine endometrial polyps, uterine fibroids, and cervical and intrauterine adhesions, respectively.

Comparisons of results of hysteroscopy, vaginal ultrasound, and pathological examinations are shown in Figure 6. There were certain differences between the diagnostic results of simple hysteroscopy or simple vaginal ultrasound and the clinical-pathological results. This suggested that there were some misdiagnoses in the single hysteroscopy as well as a vaginal ultrasound of patients with endometrial hyperplasia, endometritis, endometrial polyps, uterine fibroids, and cervical and intrauterine adhesions.

**Figure 7:**
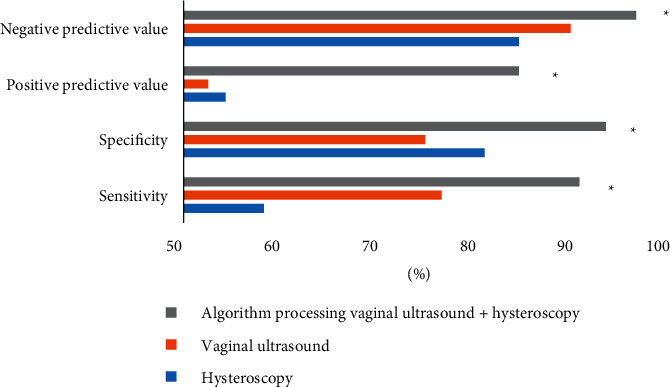
Comparison of the diagnostic accuracy of different examination methods. ^*∗*^indicates that there was a statistically significant difference compared with simple hysteroscopy or a vaginal ultrasound (*P* < 0.05).

Figure 7 is a comparison chart of the diagnostic accuracy of different inspection methods. The results showed that, compared with single hysteroscopy or vaginal ultrasound, the vaginal ultrasound under the improved *K*-means clustering color image segmentation algorithm combined with hysteroscopy had significantly increased sensitivity, specificity, negative likelihood ratio, and positive likelihood ratio (90.5%, 93.2%, 84.3%, and 96.3%). The data of each item were significantly different, which was of statistical significance (*P* < 0.05).

## 4. Discussion

AUB is a common gynecological symptom and physical sign of abnormal bleeding originating from the uterine cavity. AUB needs to exclude not only bleeding related to pregnancy and puerperium but also prepubertal and postmenopausal bleeding; it is limited to nonpregnant women of childbearing age [18]. Clinically, in the examination of patients with AUB, the systemic examination and gynecological examination are indispensable at the initial diagnosis. Relevant signs, such as sexual characteristics, height, lactation, body weight, body hair, and abdominal mass, can be found in time. It is helpful to determine the source of bleeding, exclude cervix and vaginal lesions, and find abnormal uterine structure. Combined with necessary auxiliary examinations, the etiology of AUB can be determined [19]. In clinical practice, vaginal ultrasound is often used in the diagnosis of AUB patients. Its advantages are high resolution, more accurate examination results, no need to hold a full bladder, and fewer interference factors [20]. Vaginal ultrasound is to put the vaginal probe on a condom and insert it into the vagina for examination. The probe is relatively close to the uterus and accessories, and the image resolution is high, which is allowed to observe the situation in the uterus, accessories, and pelvic cavity more clearly [21]. For example, the gestational sac can be observed earlier than through the transabdominal B-ultrasound, the growth of follicles can be monitored, the blood flow of the endometrium can be better known, and the endometrium can be better graded [22]. Vaginal ultrasound does not require the patients to hold a full bladder, so it saves time; it is also not affected by the patients' abdominal fat hypertrophy. However, vaginal ultrasound will be affected by the amount of bleeding in the diagnosis of AUB, and there are certain cases of missed diagnosis and misdiagnosis [23, 24].

To improve some problems existing in vaginal ultrasound examination, the *K*-means algorithm was specially introduced to improve the clustering color image segmentation algorithm, hoping to further improve the diagnostic accuracy of vaginal ultrasound for clinical abnormal uterine bleeding. The results showed that compared with the traditional *K*-means clustering algorithm, the running time of the *K*-means improved clustering color image segmentation algorithm was significantly shortened in training sets 1, 2, 3, 4, and 5 and clinical vaginal ultrasound images, and the MSE was significantly decreased, while the PSNR was significantly increased, with statistically significant differences (*P* < 0.05). Therefore, the *K*-means improved clustering color image segmentation algorithm can not only significantly improve the image segmentation effect but also significantly shorten the ultrasonic image processing time and further improve the efficiency of clinical disease diagnosis. In this study, *K*-means was used to improve the clustering color image segmentation algorithm and obtain relatively accurate initial parameters of the algorithm through the image HSI color space adaptive, which can significantly reduce the requirements on initial input parameters of the image. The method of computing the similarity of each pixel using multidimensional feature vector can greatly reduce the misclassification rate of each pixel in the image, thus reducing the number of iterations during the operation of the algorithm, and improving the image segmentation efficiency while maintaining low time complexity [25, 26]. *K*-means improved clustering color image segmentation algorithm before and after processing abnormal uterine bleeding patient vaginal ultrasound image display. After processed by *K*-means improved clustering color image segmentation algorithm, the visual texture of the patient's vaginal ultrasound image was clearer, the color segmentation of each pixel was clearer, and the contour imaging of the lesion site in the patient's uterus was more continuous. The results of hysteroscopy, vaginal ultrasound, and pathological examination showed that there were certain differences between the diagnosis results of hysteroscopy alone or the results of vaginal ultrasound alone and the clinicopathological results. There were certain misdiagnoses in patients with endometrial hyperplasia, endometritis, endometrial polyps, uterine fibroids, and cervical and intrauterine adhesions, as well as in patients with pure hysteroscopy and pure vaginal ultrasound, which were consistent with the research results of Sauvan et al. [27]. Different ways of checking the diagnostic accuracy comparison results showed that compared with the pure hysteroscopic examination or vaginal ultrasound examination, the diagnosis sensitivity, specificity, negative likelihood ratio, and positive likelihood ratio of the *K*-means improved clustering color image segmentation algorithm under the treatment of vaginal ultrasound combined hysteroscopy in patients with abnormal uterine bleeding were all greatly improved. There were significant differences among all groups (*P* < 0.05). This indicates that the combination of vaginal ultrasound and hysteroscopy based on the *K*-means improved clustering color image segmentation algorithm can significantly improve the clinical diagnostic accuracy of abnormal uterine bleeding diseases and reduce misdiagnosis and missed diagnosis caused by ultrasonic image bleeding volume interference, noise problems, and other reasons. However, the related optimization procedures of the *K*-means improved clustering color image segmentation algorithm are very complex and need to be further studied.

## 5. Conclusion

In conclusion, the combination of vaginal ultrasound imaging and hysteroscopy based on the *K*-means improved clustering color image segmentation algorithm can significantly improve the clinical diagnostic accuracy of patients with abnormal uterine bleeding, which provides a certain reference value for the improvement of clinical diagnosis and treatment efficiency of patients with abnormal uterine bleeding. In this study, based on the simple hysteroscopy and vaginal ultrasound, the *K*-means improved clustering color image segmentation algorithm was introduced to perform intelligent optimization processing on vaginal ultrasound images of patients with abnormal uterine bleeding. However, there are still some shortcomings in this study. There are too few evaluation indexes introduced to discuss the processing performance of the *K*-means improved clustering color image segmentation algorithm, which fails to comprehensively evaluate the adaptive performance of the algorithm. In addition, the types of patients with abnormal uterine bleeding included in this study are not very comprehensive, and the sample size is also small, so more and more comprehensive samples are expected to be included for further study.

## Figures and Tables

**Table 1:** Parameter information.

Hysteroscopy	Inner diameter	3 mm
	External diameter	5 mm

Distention media	Normal saline	

Hysteroscopy parameter	Distention pressure	110–139 mmHg
	Electric coagulation power	60 W
	Cutting power	90 W
	Velocity of dilatation medium	300 ml/min

## Data Availability

The data used to support the findings of this study are available from the corresponding author upon request.
